# Seroprevalence of IgG and Subclasses against the Nucleocapsid of SARS-CoV-2 in Health Workers

**DOI:** 10.3390/v15040955

**Published:** 2023-04-13

**Authors:** Karen Cortés-Sarabia, Kenet Hisraim Palomares-Monterrubio, Jesús Omar Velázquez-Moreno, Víctor Manuel Luna-Pineda, Marco Antonio Leyva-Vázquez, Amalia Vences-Velázquez, Roberto Dircio-Maldonado, Oscar Del Moral-Hernández, Berenice Illades-Aguiar

**Affiliations:** 1 Laboratorio de Inmunobiología y Diagnóstico Molecular, Facultad de Ciencias Químico Biológicas, Universidad Autónoma de Guerrero, Chilpancingo de los Bravo 39086, Mexico; 2 Unidad de Investigación en Inmunología y Proteómica, Laboratorio de Investigación en COVID-19, Hospital Infantil de México “Federico Gómez”, Mexico City 06720, Mexico; 3 Laboratorio de Biomedicina Molecular, Facultad de Ciencias Químico Biológicas, Universidad Autónoma de Guerrero, Chilpancingo de los Bravo 39086, Mexico; 4 Laboratorio de Virología, Facultad de Ciencias Químico Biológicas, Universidad Autónoma de Guerrero, Chilpancingo de los Bravo 39086, Mexico

**Keywords:** N protein, SARS-CoV-2, COVID-19, health workers, antibodies, IgG

## Abstract

Background: The nucleocapsid protein of SARS-CoV-2 participates in viral replication, transcription, and assembly. Antibodies against this protein have been proposed for the epidemiological analysis of the seroprevalence of COVID-19 associated with natural infection by SARS-CoV-2. Health workers were one of the most exposed populations, and some had an asymptomatic form of the disease, so detecting IgG antibodies and subclasses against the N protein can help to reclassify their epidemiological status and obtain information about the effector mechanisms associated with viral elimination. Methods: In this study, we analyzed 253 serum samples collected in 2021 and derived from health workers, and evaluated the presence of total IgG and subclasses against the N protein of SARS-CoV-2 by indirect ELISA. Results: From the analyzed samples, 42.69% were positive to anti-N IgG antibodies. A correlation between COVID-19 asymptomatic infection and IgG antibodies was observed (*p* = 0.006). The detected subclasses were: IgG1 (82.4%), IgG2 (75.9%), IgG3 (42.6%), and IgG4 (72.6%). Conclusions: This work provides evidence about the high seroprevalence of total IgG and subclasses of anti-N and their relations with the asymptomatic infection of SARS-CoV-2 and related symptoms.

## 1. Introduction

SARS-CoV-2 is an enveloped single-strand RNA (+ssRNA) virus [1] that encodes for 16 non-structural proteins (nsp 1-16), nine accessory proteins, and four structural proteins: spike (S), envelope (E), membrane (M), and nucleocapsid (N) [2,3]. The spike protein has been proposed as a therapeutic target due to its function in binding to ACE2 (angiotensin-converting enzyme 2) and host cell infection [4,5,6], whereas the N protein is associated with viral replication/transcription, the formation and maintenance of the ribonucleoprotein complex (RNP) [7], the assembly of virions by the interaction with the M protein, and the regulation of the viral cycle in the host cell [8]. The N protein can induce cellular and humoral immune responses [9]. IgM and IgG antibodies against N can be detected during the first ten days after the onset of symptoms and are associated with early infection [10]. This protein is highly conserved in SARS-CoV-2 and possesses less cross-reactivity with other Coronaviruses [11,12], and has fewer mutations than spike protein, which allows the detection of antibodies against other variants of concern (VOCs) such as B.1.351 (Beta), P.1 (Gamma), B.1.617.2 (Delta) and B.1.1.529 (Omicron) [12].

The prevalence of IgG antibodies against the N protein varied according to the geographic region, disease severity, group, and occupational exposition [13,14,15]; specifically in health workers, the reported prevalence went from 4.14% to 41.7% [14,16,17,18]. Health workers had a higher risk of infection due to direct contact with infected patients and the development of asymptomatic disease [19]. The IgG is the most abundant immunoglobulin in the bloodstream. It contributes to the immune response by neutralizing viruses, activating the complement system, and binding to Fcγ receptors [20,21]. Four subclasses have been described (IgG1, IgG2, IgG3, and IgG4), and each subclass has different biological activities [20]. Functionally, IgG1 and IgG3 are potent inductors of immunological mechanisms mediated by the Fc, as are IgG2 and IgG4, and their evaluation can provide evidence about the immunological mechanisms associated with the elimination of SARS-CoV-2 [20,22]. The presence of IgG1 and IgG3 has been described in the general population [23,24,25]. It is essential to evaluate the presence of IgG and subclasses against the N protein in health workers due to the high risk of dissemination associated with direct contact with COVID-19 patients [19,26]. This study aimed to evaluate the prevalence of IgG and subclasses in serum samples derived from health workers, and provide biological, clinical, and epidemiological data about symptomatic or asymptomatic infection with SARS-CoV-2 in this population.

## 2. Materials and Methods

### 2.1. Study Design and Sample Collection

A total of 253 serum samples were collected from health workers of the general hospital “Dr. Raymundo Abarca Alarcón”. Samples were collected on January 2021 (before receiving the first COVID-19 vaccine) and stored at −20 °C until use. A survey was used to collect clinical and epidemiological data and included age, workplace, COVID-19, diagnostic method (RT-PCR, antigen, or clinical), symptomatology (fever, headache, loss of smell, loss of taste, breathlessness, chest pain, cough, sore throat, burning eyes, congested nose, muscle pain, fatigue, chills, sweating, vomit, and diarrhea), number of times diagnosed with COVID-19, and comorbidities (obesity, asthma, neurological disease, liver disease, and hypertension).

### 2.2. Indirect ELISA for Total IgG

As an antigen, we used the recombinant N protein of SARS-CoV-2 produced in *Escherichia coli*. The process was carried out as previously described by our workgroup, with some modifications [27]. First, microtiter plates (Sigma-Aldrich) were coated with 100 µL/well of recombinant N protein to a final concentration of 0.1 µg/mL in a coating buffer (50 mM Na_2_CO_3_/NaCO_3_H, pH 9.6). The plates were incubated for 1 h at 37 °C and then blocked for 30 min at 37 °C with 200 µL/well of 5% skimmed milk diluted in phosphate-buffered saline (PBS)-Tween 20 (0.05%). For the primary antibody, 100 µL/well of serum samples (1:50 dilution) by duplicate were incubated for 30 min at 37 °C. Later, 100 µL/well of mouse monoclonal anti-human IgG (Millipore 1:10,000 dilution) was added for 45 min and incubated at 37 °C. Finally, 100 µL/well of anti-mouse H + L coupled to horseradish peroxidase (HRP) (Invitrogen 1:10,000 dilution) was added for 45 min at 37 °C. After every step, the plates were washed thrice with 200 μL/well of PBS-Tween 20 0.05% for 5 min. The enzymatic reaction was developed using o-phenylenediamine dihydrochloride (Sigma-Aldrich) and stopped using 2 N H_2_SO_4_. The optical density (OD) was measured at 492 nm using a microplate reader. Samples with OD >0.250 were considered positive (Supplementary Material Figure S1).

### 2.3. Standardization and Detection of IgG Subclasses

IgG subclasses were detected only in the 108 positive samples to total IgG. A previous standardization was carried out using negative samples collected in 2014. Mouse anti-IgG1, anti-IgG2, anti-IgG3 and anti-IgG4 monoclonal antibodies were used as follows: 1:1000 IgG1 (Sigma-Aldrich Cat #6775), 1:25,000 IgG2 (Sigma-Aldrich Cat #B-3398), 1:500 IgG3 (Sigma-Aldrich Cat #B-3523), and 1:1000 IgG4 (Sigma-Aldrich Cat #B-3648 1:1000). Later, we performed the same method described above (total IgG), and instead of using mouse anti-human IgG, we added 100 µL/well of each monoclonal antibody against every subclass. The mean ± five standard deviations of 88 negative samples collected during 2014 were used for the cut-off value. The cut-off value for each subclass was ≥0.070 IgG1, ≥0.175 IgG2, ≥0.100 IgG3, and ≥0.100 IgG4. The following controls were added during each evaluation: positive sample to IgG antibodies against the N protein, negative sample to IgG antibodies against the N protein (collected during 2014), blank (no antigen was fixed), and conjugate control (IgG fixed on the ELISA plate to test the secondary antibody).

### 2.4. Statistical Analysis

A database was constructed in STATA V.15 using all analyzed variables (age, sex, COVID-19 infection, symptoms, comorbidities) and results (total IgG and subclasses) from each evaluation. For non-parametric quantitative variables, we used percentile p25 and p75, and median. X^2^ or Fisher’s exact test obtained the *p* value for the qualitative variable. For the non-parametric quantitative variables, the Mann–Whitney U test was used. The heat maps and graphs were constructed using GraphPad Prism V.8.

## 3. Results

By indirect ELISA, we evaluated the prevalence of IgG against the N protein in 253 serum samples derived from health workers. Of the analyzed samples, 145 were negative (57.31%) and 108 were positive (42.69%). Negative and positive samples of IgG were associated with age, sex, COVID-19, workplace, diabetes, obesity, asthma, and hypertension. The analyzed population was around 32–45 years old; most were women (75.89%), and 81.03% (*n* = 205) were negative for infection with SARS-CoV-2. Of all the negative patients, 115 were negative for IgG and 79 were positive (*p* = 0.006). The population was mainly integrated by nurses (58.5%) and doctors (20.55%) that were in direct contact with COVID-19 patients, and only 31.56% reported obesity as a comorbidity (Table 1). Additionally, we associated COVID-19 related-symptoms such as fever, headache, loss of smell, loss of taste, breathlessness, chest pain, cough, and sore throat with the presence of IgG, in which only the loss of taste was statistically significant associated (Supplementary Material Figure S2).

**Figure 1:**
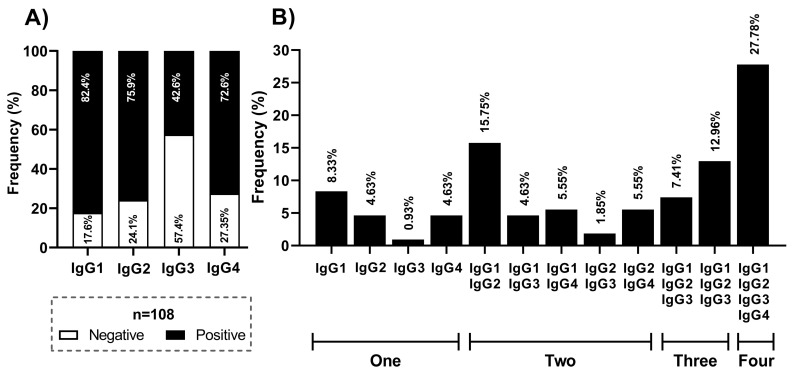
Seroprevalence of IgG subclasses against N protein of SARS-CoV-2 in the studied population. (**A**) Frequency of each subclass (IgG1 to IgG4) in the positive samples to total IgG. (**B**) Frequency of each potential combination of subclasses.

Later, we tested for IgG subclasses (IgG1-IgG4) in the positive samples (*n* = 108) to total IgG. The most prevalent subclasses were IgG1 (82.4%) and IgG2 (75.9%), followed by IgG4 (72.6%) and IgG3 (42.6%) (Figure 1a). Moreover, we analyzed the frequency of each possible combination of subclasses in the same sample. We reported more than one subclass in 81.48% of the samples (33.33% had two, 20.37% had three, and 27.78% had four). The combinations of IgG1 and IgG4 (27.78%), IgG1 and IgG3 (12.96%), IgG1 and IgG2 (15.75%), and IgG1 (8.33%) were the most frequent (Figure 1b).

**Figure 2:**
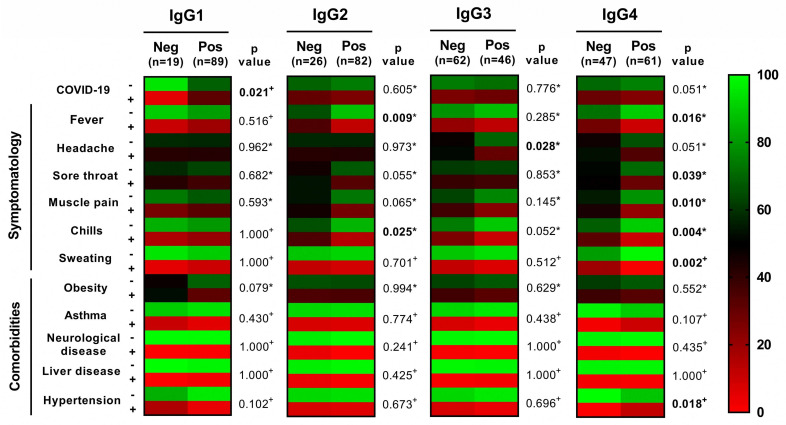
Association between IgG subclasses with COVID-19 symptoms and comorbidities. Heat map showing the frequency of symptoms, comorbidities, and COVID-19 infection in health workers in negative and positive samples to IgG subclasses. Four heat maps were constructed and correspond to each subclass. Frequency and *p*-value were added. ^+^ Fisher’s exact, * Chi2.

Finally, we constructed a heat map and associated the presence of IgG subclasses against the N protein with the diagnosis of COVID-19, symptomatology (fever, sore throat, muscle pain, chills, sweating), and comorbidities (obesity, asthma, neurological disease, liver disease, and hypertension). The presence of IgG1 was associated with a positive diagnosis of COVID-19 (*p* = 0.021), IgG2 was associated with fever (*p* = 0.009) and chills (*p* = 0.025), IgG3 was associated with headache (*p* = 0.028), and IgG4 was associated with fever (*p* = 0.016), sore throat (*p* = 0.039), muscle pain (*p* = 0.010), chills (*p* = 0.004), sweating (*p* = 0.002), and hypertension (*p* = 0.018) (Figure 2). A detailed version of the heat maps is provided in Supplementary Material Table S1.

## 4. Discussion

The virion of SARS-CoV-2 contains four structural proteins: the S (spike) protein, the M (membrane) protein, the E (envelope) protein, and the N (nucleocapsid) protein. Remarkably, the N protein is involved in packaging RNA and viral cycle regulation [7]. Antibodies against this protein are associated with moderate protection from future infection with SARS-CoV-2 [28], and provide evidence for recategorizing asymptomatic patients, differentiating vaccinated from naturally infected patients, and for epidemiological purposes. Antibodies against N are more useful for detecting and diagnosing early infection [10]. In this study, we evaluated the prevalence of IgG and subclasses against the N protein in serum samples derived from health workers, the leading group exposed to COVID-19 patients. The seroprevalence of IgG antibodies against SARS-CoV-2 varied according to the study population and geographic region. We reported a seroprevalence of 42.69% of anti-N IgG antibodies; similar studies have reported a seroprevalence from 16.9% to 41.7% [14,17,18], associated with the constant exposition of health workers to COVID-19 patients and asymptomatic infections. In our studied population, patients reported obesity, hypertension, and diabetes. These comorbidities have been associated with high titers of IgG antibodies against the S and N proteins during the convalescent phase [29]. Specifically, the correlation between asthma and the presence of IgG antibodies (*p* = 0.040) could be associated with the expression of genes related to ACE2 and infection with SARS-CoV-2 in patients with asthma, obstructive chronic disease, and hypertension [30]. Symptoms such as headache, nasal congestion, fatigue, and cough were mainly associated with the viral load [31] and infection with delta and omicron variants of concern [32]. Additionally, moderate or severe symptoms are related to a positivity of IgG antibodies and higher neutralizing activity [33].

The subclasses of IgG are associated with changes in the structure and biological functions. IgG1 activates the classical pathway of complement, phagocytosis mediated by macrophages, monocytes, and neutrophils, and antibody-dependent cellular cytotoxicity (ADCC). IgG2 cannot bind the complement and has a low affinity to FcγR, and could be an anti-inflammatory antagonist of IgG1 [34], which explains the similar reported frequencies of IgG1 and IgG2 in this study. Additionally, IgG2 recognizes protein-derived antigens and carbohydrate-derived antigens, both commonly found in SARS-CoV-2. IgG3 is a potent inflammatory inductor; however, in this study we reported a seroprevalence of 42.6%, which could be associated with a shorter half-life due to an extended hinge region and proteolytic degradation [35,36]. IgG4 has been associated with chronic exposure to the antigen [36]. In this study, the high prevalence of this subclass could be due to the direct and repeated contact with COVID-19 patients, thus promoting the class switch to IgG4. Previous studies have evaluated the presence of IgG subclasses’ anti-RBD, and reported that the most prevalent subclasses were IgG2 (60.47%) and IgG1 (53.49%), followed by IgG3 (44.19%) and IgG4 (2.33%) during the first 30 days of infection [37], whereas specifically against N protein, a seroprevalence of 70% to IgG1, 67% to IgG3, 52% to IgG2, and 45% to IgG4 during the first 7–62 days post-infection has been reported [38].

Finally, we performed associations between the symptoms associated with COVID-19 and subclasses: we found an inverse correlation between the presence of IgG2 and fever, IgG3 and headache, and IgG4 with fever, headache, sore throat, muscular pain, chills, and sweating. Symptoms are commonly associated with the presence of IgG1, IgG3, and IgG2 [24,25], and with the severity of the disease. Future studies need to be performed in other populations and age groups to provide epidemiological data about the natural infection of SARS-CoV-2 by searching IgG antibodies’ anti-N. Additionally, complementary analysis, including the quantification of IgG subclasses, should be addressed to understand the seroprevalence of anti-N IgG antibodies and the biological functions promoted by each subclass.

## 5. Conclusions

This study provides evidence of the high prevalence of IgG (46.69%) and the presence of the four subclasses of IgG against the N protein in samples derived from one of the most exposed risk groups to COVID-19 infection and dissemination. Subclasses were associated with the asymptomatic infection of SARS-CoV-2 and symptoms such as fever, chills, headache, sore throat, muscle pain, sweating, and hypertension. The evaluation of IgG and subclasses against the N protein is essential to understand the humoral immune response against SARS-CoV-2, and to describe the effector mechanisms promoted by each subclass.

## Figures and Tables

**Table 1:** IgG seroprevalence in the studied population.

Characteristics *n* (%)	Total (*n* = 253)	IgG Negative (*n* = 145)	IgG Positive (*n* = 108)	*p* Value
**Age** (Median p25–p75)	38 (32.5–43.5)	39 (33–43)	38 (32–44.5)	0.7478 °
Sex				
Female	192 (75.89)	115 (79.31)	77 (71.30)	0.140 *
Male	61 (24.11)	30 (20.69)	31 (28.70)	
COVID-19				
Negative	205 (81.03)	126 (86.90)	79 (73.15)	0.006 *
Positive	48 (18.97)	19 (13.10)	29 (26.85)	
Workplace				
Doctor	52 (20.55)	27 (18.62)	25 (23.15)	0.286 *
Nurse	148 (58.50)	92 (63.45)	56 (51.85)	
Stretcher-bearer	14 (5.53)	6 (4.14)	8 (7.41)	
Administrative	16 (6.32)	9 (6.21)	7 (6.48)	
Dentist	2 (0.79)	0 (0.00)	2 (1.84)	
Psychologist	6 (2.37)	2 (1.38)	4 (3.70)	
Social worker	9 (3.56)	6 (4.14)	3 (2.78)	
User service	1 (0.40)	0 (0.00)	1 (0.93)	
Laboratory	4 (1.58)	3 (2.06)	1 (0.93)	
Physiotherapist	1 (0.40)	0 (0.00)	1 (0.93)	
Diabetes				
Negative	241 (95.26)	139 (95.86)	102 (94.44)	0.600 *
Positive	12 (4.74)	6 (4.14)	6 (5.56)	
Obesity				
Negative	174 (68.77)	104 (71.72)	70 (64.81)	0.241 *
Positive	79 (31.23)	41 (28.28)	38 (35.19)	
Asthma				
Negative	244 (96.44)	143 (98.62)	101 (93.52)	0.040 **^+^**
Positive	9 (3.56)	2 (1.38)	7 (3.56)	
Hypertension				
Negative	233 (92.09)	132 (91.03)	101 (93.52)	0.469 *
Positive	20 (7.91)	13 (8.97)	7 (6.48)	

Symbology: ° Mann–Whitney *U*, **^+^** Fisher’s exact, * Chi^2^.

## Data Availability

Data are contained within the article or supplementary material.

## Supplementary Materials

The following supporting information can be downloaded at: https://www.mdpi.com/article/10.3390/v15040955/s1, Figure S1: Schematic representation of the indirect ELISA protocol used in this study. Figure S2: Symptomatology and its association with the presence of IgG anti-N antibodies. Table S1: Association of subclasses with symptomatology and comorbidities.
