# Supplementary material for: Seroprevalence of IgG and Subclasses against the Nucleocapsid of SARS-CoV-2 in Health Workers
Source: Viruses. 2023 Apr 13;15(4):955. doi: 10.3390/v15040955 (PMC10141201; doi:10.3390/v15040955)
Supplement: Supplementary file 1 [file viruses-15-00955-s001.zip › Figure S1.pdf]

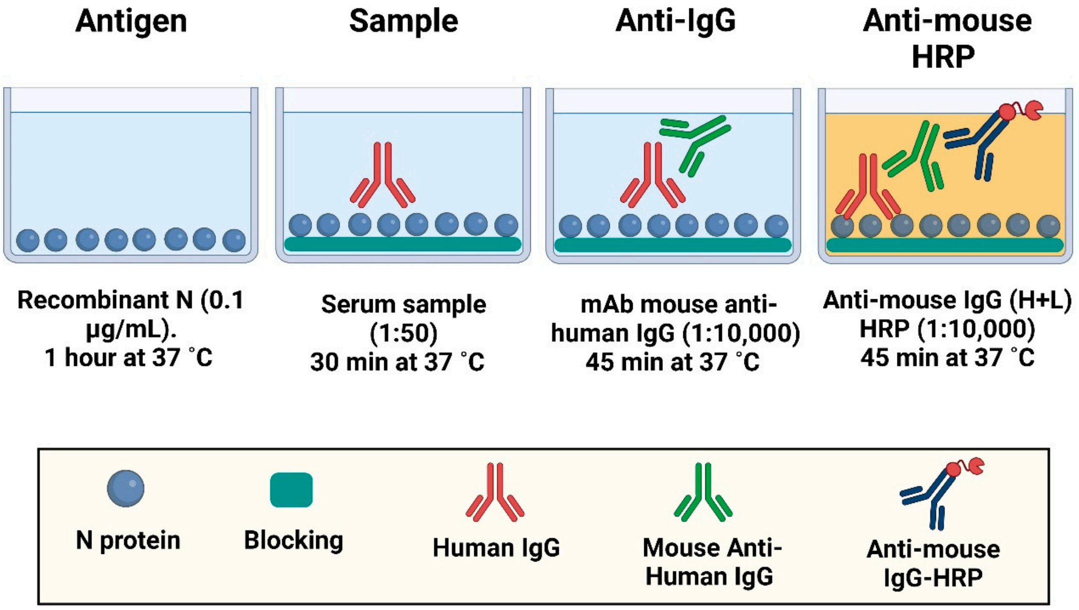

Figure S1.- Schematic representation of the indirect ELISA protocol used in this study. In this method, we used as antigen the recombinant N protein of SARS-CoV-2 to a final concentration of 0.1 µg/mL for 1 hour. The plates were blocked with 5% skim milk diluted in PBS-Tween 20 for 30 min. The sample was diluted 1:50 for total IgG and 1:25 for subclass detection in PBS and incubated for 30 min. Later, an anti-human IgG (or subclass) monoclonal antibody was added for 45 min. Finally, an anti-mouse IgG (H+L) coupled to HRP was incubated for 45 min.
