# Supplementary material for: Seroprevalence of IgG and Subclasses against the Nucleocapsid of SARS-CoV-2 in Health Workers
Source: Viruses. 2023 Apr 13;15(4):955. doi: 10.3390/v15040955 (PMC10141201; doi:10.3390/v15040955)
Supplement: Supplementary file 1 [file viruses-15-00955-s001.zip › Figure S2.pdf]

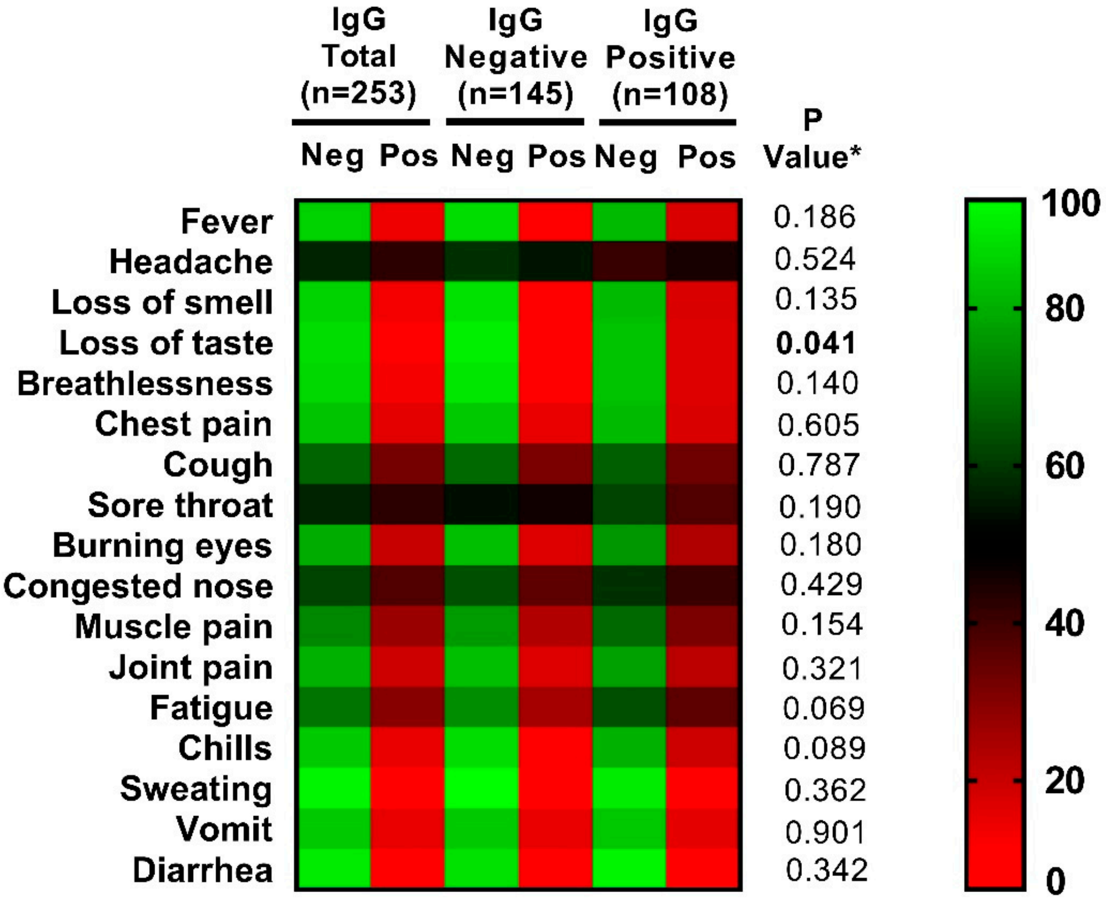

Figure S2.- Symptomatology and its association with the presence of IgG anti-N antibodies. Heat map illustrating the association of the most common symptoms of COVID-19 and the presence of IgG antibodies. P value was calculated by using chi square test.
