# Supplementary material for: Seroprevalence of IgG and Subclasses against the Nucleocapsid of SARS-CoV-2 in Health Workers
Source: Viruses. 2023 Apr 13;15(4):955. doi: 10.3390/v15040955 (PMC10141201; doi:10.3390/v15040955)
Supplement: Supplementary file 1 [file viruses-15-00955-s001.zip › Table S1.pdf]

Table S1.- Association of subclasses with symptomatology and comorbidities.

Characteristic		IgG1 n (%)		*p* value	OR	IC (95%)
		Negative	Positive			
Age^{x}		38 (30-45)	38 (32-44)	0.821^{o}	-	-
COVID-19	Negative	18 (94.74)	61 (68.54)	**0.021^{+}**	8.26	1.17-356.27
	Positive	1 (5.26)	28 (31.46)			
Fever	Negative	17 (89.47)	72 (80.90)	0.516^{+}	2.00	0.41-19.46
	Positive	2 (10.53)	17 (19.10)			
Headache	Negative	11 (57.89)	51 (57.30)	0.962*	1.02	0.34-3.24
	Positive	8 (42.11)	38 (42.70)			
Sore throat	Negative	11 (57.89)	56 (62.92)	0.682*	0.81	0.27-2.58
	Positive	8 (42.11)	33 (37.08)			
Muscle ache	Negative	14 (73.68)	60 (67.42)	0.593*	1.35	0.41-5.26
	Positive	5 (26.32)	29 (32.58)			
Chills	Negative	16 (84.21)	71 (79.78)	1.000^{+}	1.35	0.33-7.99
	Positive	3 (15.79)	18 (20.22)			
Sweating	Negative	18 (94.74)	80 (89.89)	1.000^{+}	2.03	0.25-93.66
	Positive	1 (5.26)	9 (10.11)			
**Comorbidity**						
Obesity	Negative	9 (47.37)	61 (68.54)	0.079*	0.41	0.13-1.28
	Positive	10 (52.63)	28 (31.46)			
Asthma	Negative	17 (89.47)	84 (94.38)	0.430^{+}	0.51	0.08-5.77
	Positive	2 (10.53)	5 (5.62)			
Neurological diseases	Negative	19 (100)	88 (98.88)	1.000^{+}	-	-
	Positive	0 (0.00)	1 (0.93)			
Liver diseases	Negative	19 (100)	87 (97.75)	1.000^{+}	-	-
	Positive	0 (0.00)	2 (2.25)			
Hypertension	Negative	16 (84.21)	85 (95.51)	0.102^{+}	0.25	0.04-1.93
	Positive	3 (15.79)	4 (4.49)			

Symbology: ^{x}Median (p25-p75), ^{o}Mann-Whitney *U*, $^{+}$Fisher's exact, *Chi^{2}.

Characteristic		IgG2 n (%)		*p* value	OR	IC (95%)
		Negative	Positive			
**Age**ˣ		38 (32-45)	37.5 (32-44)	0.7818º	-	-
**COVID-19**	**Negative**	18 (69.23)	61 (74.39)	0.605*	0.77	0.27-2.38
	**Positive**	8 (30.77)	21 (25.61)			
**Fever**	**Negative**	17 (65.38)	72 (87.80)	**0.009***	0.56	0.16-1.78
	**Positive**	9 (34.62)	10 (12.20)			
**Headache**	**Negative**	15 (57.69)	47 (57.32)	0.973*	1.02	0.38-2.76
	**Positive**	11 (42.31)	35 (42.68)			
**Sore throat**	**Negative**	12 (46.15)	55 (67.07)	0.055*	0.42	0.15-1.14
	**Positive**	14 (53.85)	27 (32.93)			
**Muscle ache**	**Negative**	14 (53.85)	60 (73.17)	0.065*	0.43	0.16-1.19
	**Positive**	12 (46.15)	22 (26.83)			
**Chills**	**Negative**	17 (65.38)	70 (85.37)	0.025*	0.32	0.11-1.03
	**Positive**	9 (34.62)	12 (14.63)			
**Sweating**	**Negative**	23 (88.46)	75 (91.46)	0.701⁺	0.72	0.15-4.65
	**Positive**	3 (11.54)	7 (8.54)			
**Comorbidity**						
**Obesity**	**Negative**	17 (65.38)	53 (64.63)	0.994*	1.03	0.38-2.98
	**Positive**	9 (34.62)	29 (35.37)			
**Asthma**	**Negative**	24 (92.31)	77 (93.90)	0.774⁺	0.78	0.12-8.70
	**Positive**	2 (7.69)	5 (6.48)			
**Neurological diseases**	**Negative**	25 (96.15)	82 (100)	0.241⁺	-	-
	**Positive**	1 (3.85)	0 (0.00)			
**Liver diseases**	**Negative**	25 (96.15)	81 (98.78)	0.425⁺	0.31	0.004-25.19
	**Positive**	1 (3.85)	1 (1.22)			
**Hypertension**	**Negative**	24 (92.31)	77 (93.90)	0.673⁺	0.79	0.12-8.81
	**Positive**	2 (7.69)	5 (6.10)			

Symbology: ˣMedian (p25-p75), ºMann-Whitney *U*, ⁺Fisher's exact, *Chi^2.

Characteristic		IgG3 n (%)		*p* value	OR	IC (95%)
		Negative	Positive			
**Age^{x}**		37 (32-45)	39 (32-45)	0.6678^{o}	-	-
**COVID-19**	**Negative**	46 (74.19)	33 (71.74)	0.776*	1.13	0.44-2.90
	**Positive**	16 (25.81)	13 (28.26)			
**Fever**	**Negative**	49 (79.03)	40 (86.96)	0.285*	0.56	0.16-1.78
	**Positive**	13 (20.97)	6 (13.04)			
**Headache**	**Negative**	30 (48.39)	32 (69.57)	**0.028***	0.41	0.17-0.98
	**Positive**	32 (51.61)	14 (30.43)			
**Sore throat**	**Negative**	38 (61.29)	29 (63.04)	0.853*	0.93	0.39-2.18
	**Positive**	24 (38.71)	17 (36.96)			
**Muscle ache**	**Negative**	39 (62.90)	35 (76.09)	0.145*	0.53	0.20-1.34
	**Positive**	23 (37.10)	11 (23.91)			
**Chills**	**Negative**	46 (74.19)	41 (89.13)	0.052*	0.35	0.09-1.13
	**Positive**	16 (25.81)	5 (10.87)			
**Sweating**	**Negative**	55 (88.71)	43 (93.48)	0.512^{+}	0.55	0.09-2.59
	**Positive**	7 (11.29)	3 (6.52)			
**Comorbidity**						
**Obesity**	**Negative**	39 (62.90)	31 (67.39)	0.629*	0.82	0.34-1.97
	**Positive**	23 (37.10)	15 (32.61)			
**Asthma**	**Negative**	57 (91.94)	44 (95.65)	0.438^{+}	0.51	0.05-3.37
	**Positive**	5 (8.06)	2 (4.35)			
**Neurological diseases**	**Negative**	61 (98.39)	46 (100)	1.000^{+}	-	-
	**Positive**	1 (1.61)	0 (0.00)			
**Liver diseases**	**Negative**	61 (98.39)	45 (98.15)	1.000^{+}	1.36	0.02-108.12
	**Positive**	1 (1.61)	1 (2.17)			
**Hypertension**	**Negative**	57 (91.94)	44 (95.64)	0.696^{+}	0.55	0.05-3.45
	**Positive**	5 (8.06)	2 (4.35)			

Symbology: ^{x}Median (p25-p75), ^{o}Mann-Whitney *U*, $^{+}$Fisher's exact, *Chi^{2}.

Characteristic		IgG4 n (%)		*p* value	OR	IC (95%)
		Negative	Positive			
**Age**^{x}		38 (32-45)	37 (31-44)	0.4566^{o}	-	-
**COVID-19**	**Negative**	33 (70.21)	46 (75.41)	0.051*	0.77	0.300-1.98
	**Positive**	14 (29.79)	15 (24.59)			
**Fever**	**Negative**	34 (72.34)	55 (90.16)	**0.016***	0.28	0.08-0.991
	**Positive**	13 (27.66)	6 (9.84)			
**Headache**	**Negative**	22 (46.81)	40 (65.57)	0.051*	0.46	0.20-1.08
	**Positive**	25 (53.19)	21 (34.43)			
**Sore throat**	**Negative**	24 (51.06)	43 (70.49)	**0.039***	0.43	0.18-1.04
	**Positive**	23 (48.94)	18 (29.51)			
**Muscle ache**	**Negative**	26 (55.32)	48 (78.69)	**0.010***	0.33	0.13-0.84
	**Positive**	21 (44.68)	13 (21.31)			
**Chills**	**Negative**	32 (68.09)	55 (90.16)	**0.004***	0.23	0.07-0.72
	**Positive**	15 (31.91)	6 (9.84)			
**Sweating**	**Negative**	38 (80.85)	60 (98.36)	**0.002**$^{+}$	0.70	0.002-0.55
	**Positive**	9 (19.15)	1 (1.64)			
**Comorbidity**						
**Obesity**	**Negative**	29 (61.70)	41 (67.21)	0.552*	0.79	0.33-1.88
	**Positive**	18 (38.30)	20 (32.79)			
**Asthma**	**Negative**	46 (97.87)	55 (90.16)	0.107^{+}	5.02	0.57-235.70
	**Positive**	1 (2.13)	6 (9.84)			
**Neurological diseases**	**Negative**	46 (97.87)	61 (100)	0.435^{+}	-	-
	**Positive**	1 (2.13)	0 (0.00)			
**Liver diseases**	**Negative**	46 (97.87)	60 (98.36)	1.000^{+}	0.76	0.01-61.47
	**Positive**	1 (2.13)	1 (1.64)			
**Hypertension**	**Negative**	47 (100)	54 (88.52)	**0.018**$^{+}$	-	-
	**Positive**	0 (0.00)	7 (11.48)			

Symbology: ^{x}Median (p25-p75), ^{o}Mann-Whitney *U*, $^{+}$Fisher's exact, *Chi^{2}.
